# Evaluation of medication-related problems in liver transplant recipients with and without an outpatient medication consultation by a clinical pharmacist: a cohort study

**DOI:** 10.1007/s11096-022-01423-6

**Published:** 2022-09-13

**Authors:** Midas B. Mulder, B. Doga, S. D. Borgsteede, A. M. van den Burg, H. J. Metselaar, C. M. den Hoed, N. G.M. Hunfeld

**Affiliations:** 1 Department of Hospital Pharmacy, Erasmus MC, University Medical Center Rotterdam, Rotterdam, The Netherlands; 2 Department of Clinical Decision Support, Health Base Foundation, Houten, The Netherlands; 3 Department of Gastroenterology and Hepatology, Erasmus MC, University Medical Center Rotterdam, Rotterdam, The Netherlands; 4 Department of Intensive Care, Erasmus MC, University Medical Center Rotterdam, Rotterdam, The Netherlands; 5 Erasmus MC Transplant Institute, University Medical Center Rotterdam, Rotterdam, The Netherlands; 6 Department of Pharmacy, Erasmus MC, University Medical Center Rotterdam, PO Box 2040, 3015 GD Rotterdam, The Netherlands

**Keywords:** Clinical pharmacy, Liver transplantation, Medication-related problems, Medication consultation, Medication safety, Medication review

## Abstract

**Background:**

Transplant recipients undergo significant changes in their medication regimen during follow-up and are at an increased risk for medication-related problems (MRPs).

**Aim:**

This study aimed to compare the prevalence and types of MRPs and interventions in liver transplant recipients with and without an outpatient medication consultation by a clinical pharmacist as well as the satisfaction with information about medicines and medication adherence.

**Method:**

We performed a single-center, observational cohort study. A retro- and prospective cohort were used and subdivided in a group that did and did not receive a medication consultation. The prevalence and types of MRPs and interventions were identified and categorized. The satisfaction parameters were evaluated using validated questionnaires.

**Results:**

Included were 291 patients. In total, 368 MRPs were identified in 197 patients in the non-medication consultation cohort (median 1; range 1–3 per patient) and 248 MRPs in 94 patients in the medication consultation cohort (median 2; range 1–4 per patient). In the medication consultation cohort, significantly fewer MRPs as unnecessary drugs (17.3% *versus* 58.7%, p < 0.001), suboptimal therapy (2.4% *versus* 9.5%, p < 0.001), untreated indication (2.8% *versus* 6.8%, p = 0.040) and underdosed drugs (0.4% *versus* 6.3%, p < 0.001) were identified. In the non-medication consultation cohort significantly more patients used unnecessary drugs (72.1% *versus* 39.4%, p < 0.001) compared to the medication consultation cohort. Patients in both cohorts are satisfied with the information about medicines and reported a high medication adherence.

**Conclusion:**

Patients in the medication consultation cohort had significantly fewer MRPs and used significantly less unnecessary drugs. Including a clinical pharmacist to the post-transplant care has an added value.

**Supplementary information:**

The online version contains supplementary material available at 10.1007/s11096-022-01423-6.

## Impact statements

- Transplant recipients undergo significant changes in their medication regimen during follow-up resulting in an increased risk for medication-related problems.
- Including an outpatient medication consultation by a clinical pharmacist in the post-transplant care results in significantly fewer medication-related problems as unnecessary drugs, suboptimal therapy, untreated indications and underdosed drugs.
- Since clinical pharmacists bring different perspectives to the post-transplant care, including one in the multidisciplinary transplant team has an added value for improving the pharmaceutical care, keeping the post-transplant care sustainable and optimizing medication safety in these patients.

## Introduction

Liver transplantation (LT) has become a lifesaving treatment option for patients with end-stage liver disease, hepatocellular carcinoma and acute liver failure [1]. Over the past decades significant developments have been made in the field of LT, which steadily led to improved outcomes and long-term survival [1, 2]. However, long-term care after LT remains complex. LT recipients undergo significant changes in their medication regimen during follow-up with an increased risk for medication-related problems (MRPs).

LT recipients need to adhere to difficult and complex therapeutic regimens[2–5]. In addition, LT recipients will usually receive more medication over the years due to the development of new-onset diabetes mellitus, hypertension, and hyperlipidemia [6, 7]. The addition of more medication could cause MRPs and possibly result in preventable drug-related hospital admissions [8, 9]. A study by Repp et al. reported that 40% of the hospital admissions following cardiac transplantation were drug-related of which 58% was preventable [10].

Hepatologists focus mainly on the liver-related problems and transplant-specific complications. Clinical pharmacists involved in the transplant care provide a broad range of different services in order to prevent MRPs such as therapeutic drug monitoring, educational activities, management of adverse drug reactions (ADRs), dosing issues and therapy optimizations [11].

Recently, we showed the added value of an outpatient monitoring program for LT recipients by a clinical pharmacist through signaling relevant discrepancies and MRPs [12]. However, no studies have been done to compare the prevalence of MRPs and interventions in patients with and without an outpatient medication consultation (MC) by a clinical pharmacist.

### Aim

This study aimed to compare the prevalence and types of MRPs and interventions in LT recipients with and without an outpatient pharmacy consultation by a clinical pharmacist as well as the satisfaction with information about medicines and medication adherence.

### Ethics approval

A waiver was given by the Medical Ethics Committee of the Erasmus University Medical Center (MEC-2019-0784). Patient data were sampled and stored in accordance with privacy regulations.

## Method

### Study design and setting

We performed an observational cohort study at the Erasmus MC Transplant Institute, University Medical Center Rotterdam, the Netherlands. For our primary objective, we retrospectively collected data between July-December 2020. For the secondary objectives, we prospectively included a second cohort of LT recipients to participate in a questionnaire study in the period March-May 2021. Both cohorts consisted of adult LT recipients > 1 year after LT who were scheduled for an annual, multidisciplinary medical check-up at the outpatient clinic. A retro- and prospective cohort were used due to practical and feasibility reasons. The use of questionnaires to evaluate the satisfaction with information about medicines and medication adherence was not regularly done in our clinic. Therefore, the prospective part of this research was performed during a research internship. No differences in the treatment protocol occurred during both periods.

At the start of this study in July 2020, 746 LT recipients were in active follow up after transplantation at the Erasmus MC. Since 2018, a clinical pharmacist has an active role in the annual, multidisciplinary medical check-up of LT recipients by conducting MCs. Detailed information about the content of the MCs by the clinical pharmacist has been previously reported [12]. All check-ups are performed on two weekdays, with the clinical pharmacist only participating on one of these days. This has resulted in two cohorts: a cohort that did not receive a MC (non-MC cohort) and a cohort that did receive a MC (MC cohort). In the non-MC cohort more LT recipients are being seen since more hepatologists have outpatient visits on that day of the week. All findings during the check-ups were registered in the patients’ medical records for further follow-up.

### Data collection

#### MRPs and interventions

For the analysis of the primary objective the following baseline characteristics were obtained from the patient medical record: age, gender, indication for liver transplantation, time after transplantation, information about re-transplantation, comorbidities, and number and type of drugs. MRPs and interventions in the non-MC cohort were identified by reviewing all information. This information included the patients’ medical history and laboratory results, such as electrolytes, renal function, and blood glucose levels. Medication reconciliation was performed in the MC cohort. MRPs and interventions identified by the clinical pharmacist as well as MRPs solved by the clinical pharmacist in the MC cohort were documented in the patients’ medical records.

#### Assessment of MRPs and interventions

For the non-MC cohort, MRPs were assessed by a pharmacological review based on all available information in the patients’ medical record after the LT recipients were seen by a hepatologist. The follow-up and corrections of the detected MRPs in the non-MC cohort was beyond the scope of this research. Two researchers independently identified these MRPs and interventions proposed by the hepatologist and categorized them into predefined categories using the Pharmaceutical Care Network Europe (PCNE) Classification V9.0 (full classification in Supplementary Table 1) [8]. Next, the classifications were compared and when dissensus occurred, both researchers reviewed their classifications and discussed these until consensus was reached.

For the MC cohort, the two researchers independently categorized the identified MRP and proposed interventions by the clinical pharmacist as registered in the patients’ medical record into the predefined categories using the PCNE Classification V9.0 [8]. Only one intervention could have been proposed for each identified MRP.

#### Satisfaction with Information about Medicines and the Medication Adherence

LT recipients in the second cohort were asked to fill out two questionnaires (translated and validated into Dutch) after their annual medical check-up: the Satisfaction with Information about Medicines (SIMS) and the Medication Adherence Reporting Scale (MARS-5) surveys [13, 14]. Besides, patients were asked to report their age, gender and highest reached educational level. The International Standard Classification of Education 2011 (ISCED 2011) was used to convert the Dutch educational system into an international one [15].

#### Assessment of the Satisfaction with Information about Medicines and the Medication Adherence

The SIMS assesses patients’ satisfaction with 17 items of information considered essential for safe and accurate self-management of medicines according to the recommendations of the Association of the British Pharmaceutical Industry (full survey in Section S1 in the Supplementary Appendix) [13, 14]. For each item, patients indicate if the information they have received is “too much,” “about right,” “too little,” “none received,” or “none needed.” Reports of “about right” and “none needed” are classified as satisfied and receive a score of 1. The remaining options are classified as dissatisfied and are scored as 0. The scores are summed up to obtain a satisfaction rating for the total scale ranging from 0 to 17. Higher summary scores indicate a higher degree of satisfaction with information received [16]. A score of ≥ 13 was interpreted as a satisfied patient and a number < 13 was interpreted as a dissatisfied patient. No threshold for satisfied *versus* dissatisfied was described in the literature. Therefore, an arbitrary threshold in which > 75% of the items of the questionnaire received a score of 1 was chosen by the researchers.

The MARS-5 compromises five short adherence statements (full survey is provided in Section S2 in the Supplementary Appendix) [14]. The MARS-5 survey was scored on a Likert-type scale ranging from “always”, “often”, “sometimes”, “rarely” to “never”. The point spread from 1 point for “always” to 5 points for “never”. A total adherence rate was obtained for each patient. A total of 25 could be achieved with higher scores indicating higher reported adherence.

### Statistical analysis

No formal sample size calculation was performed. We included every patient seen for their annual check-up in the study period at the outpatient clinic in the analysis. Variables were described using counts (%) for nominal and ordinal variables and mean (standard deviation, SD) or median (inter-quartile range, IQR) for the continuous variables, depending on the shape of the distribution.

The primary and secondary endpoints were analyzed using Pearson’s Chi-square test or Fisher’s exact tests. The latter was used in the case of a low observed count (< 10) in at least one of the cohorts. For all statistical tests, a two-sided p-value of < 0.05 was considered to indicate statistical significance. Missing values < 5% were considered as missing completely at random.

Statistical analysis was performed using SPSS for Windows, version 25.0 (SPSS, Chicago, IL).

## Results

### Primary endpoint: prevalence of and interventions proposed for MRPs

Between 30/06/2020–31/12/2020 a total of 291 LT recipients had their annual, medical check-up; 197 in the non-MC cohort and 94 in the MC cohort. Two LT recipients in the MC cohort did not show up at their annual, medical check-up.

Table 1 shows the baseline clinical and demographical characteristics of the participants. LT recipients in the non-MC cohort had a significantly higher occurrence of renal disorder as comorbidity (p < 0.001). No significant differences were found in the number of drugs on the medication list during consultation during the annual check-up (p = 0.276).

**Table 1:** Baseline clinical and demographical characteristics of LT recipients with and without an MC

	non-MC cohort (n = 197)	MC cohort (n = 94)	p-value
**Age (year) (median [IQR])**	60.0 (49.0–68.0)	60.0 (51.0–68.0)	0.455^Ω^
**Gender**			
Male (n, %)	120 (60.9%)	50 (53.2%)	0.211
**Indication liver transplantation** ^¥^			
Viral hepatitis	43 (21.8%)	29 (30.9%)	0.077
Primary sclerosing cholangitis	45 (22.8%)	21 (22.3%)	0.924
Hepatocellular carcinoma	46 (23.4%)	9 (9.6%)	0.006^∫^*
Alcohol-related liver disease	32 (16.2%)	9 (9.6%)	0.151^∫^
Acute liver failure	18 (9.1%)	5 (5.3%)	0.354^∫^
Biliary cirrhosis	14 (7.1%)	4 (4.3%)	0.441^∫^
Metabolic liver disease	13 (6.6%)	4 (4.3%)	0.595^∫^
Polycystic liver disease	9 (4.6%)	4 (4.3%)	1.000^∫^
Nonalcoholic Steatohepatitis	9 (4.6%)	4 (4.3%)	1.000^∫^
Cryptogenic cirrhosis	10 (5.1%)	6 (6.4%)	0.574^∫^
Other^a^	12 (6.1%)	13 (13.8%)	0.050
**Time after transplantation (year) (median [IQR])**	7.0 (4.0–12.0)	8.0 (4.0–15.0)	0.536^Ω^
**Retransplantation**			
Yes	21 (10.7%)	4 (4.3%)	0.076^∫^
**Comorbidities** ^**Ω**^			
Cardiovascular disease	128 (65.0%)	50 (53.2%)	0.054
Diabetes mellitus	57 (28.9%)	22 (23.4%)	0.321
Renal disorder	36 (18.3%)	4 (4.3%)	< 0.001^∫^*
Inflammatory bowel disease	35 (17.8%)	17 (18.1%)	0.947
Bone disease	31 (15.7%)	8 (8.5%)	0.100^∫^
Other^b^	83 (42.1%)	44 (46.8%)	0.452
None	23 (11.7%)	14 (14.9%)	0.441
**Number of drugs on medication list during consultation (median [IQR])**	6.0 (5.0–9.0)	7.0 (5.0–10.0)	0.276^Ω^

Data presented are counts (%) and differences between groups were analyzed using the Pearson’s chi-squared test unless otherwise noted

^Ω^Mann-Whitney U test; ^∫^Fisher’s exact test; ^¥^patients may have had more than one indication for liver transplantation and/or comorbidities; *indicates a statistical significant difference of p < 0.05

^a^other includes biliary atresia (n = 5), hemochromatosis (n = 4), Budd–Chiari syndrome (n = 3), Caroli disease (n = 2), Rendu-Osler-Weber (n = 2), cystic fibrosis (n = 2), Alagille syndrome (n = 1), hemangioendothelioma (n = 1), Abernethy Syndrome (n = 1), echinococcosis (n = 1), acute fatty liver of pregnancy (n = 1), Crigler–Najjar syndrome (n = 1), cholangiocarcinoma (n = 1);

^b^other includes haematological, immunological, metabolic and psychological morbidities

Abbreviations: IQR, inter-quartile range; non-MC cohort, no medication consultation cohort; MC cohort, medication consultation cohort

### Medication related problems

In total, 616 MRPs were identified: 368 in the non-MC cohort (median per patient, 1.0; IQR, 1.0–3.0) and 248 in the MC cohort (median per patient, 2.0; IQR, 1.0–4.0). Most LT recipients had at least one MRP, 173 (87.8%) in the non-MC cohort and 89 (94.7%) in the MC cohort.

Table 2 shows the prevalence and examples of the identified MRPs in both cohorts. In the MC cohort, significantly fewer MRPs as unnecessary drugs (17.3% *versus* 58.7%, p < 0.001), wrong drugs/suboptimal therapy (2.4% *versus* 9.5%, p < 0.001), untreated indication (2.8% *versus* 6.8%, p = 0.040), and too low dosed drugs (0.4% *versus* 6.3%, p < 0.001) were detected compared to the non-MC cohort. In the MC cohort significantly more MRPs as unintentional nonadherence (9.0% *versus* 0.0%, p < 0.001), problems in drug use (24.2% *versus* 1.6%, p < 0.001), questions regarding the drugs (4.0% *versus* 0.0%, p < 0,001), and other problems (28.2% *versus* 3.0%, p < 0.001) were detected compared to the non-MC cohort.

**Table 2:** Prevalence and examples of the identified MRPs in LT recipients with and without an MC

MRPs	N (%) instances of MRPs in non-MC cohort (n = 368)	N (%) instances of MRPs in MC cohort (n = 248)	p-value	Example of MRPs
Nonadherence				
Intentional	0 (0.0%)	3 (1.2%)	0.065	Nonadherence with magnesium gluconate due to diarrhea as an adverse drug reaction.
Unintentional	0 (0.0%)	9 (3.6%)	< 0.001*	Nonadherence with ursodeoxycholic acid due to forgetting to take the drug during the holiday.
Adverse drug reaction	34 (9.2%)	31 (12.5%)	0.196^Ω^	Hypomagnesaemia when using a proton pump inhibitor.
Drug interaction	0 (0.0%)	2 (0.8%)	0.162	Use of clindamycin cure during a holiday, which causes an interaction with cyclosporine.
Indication				
Wrong drug/suboptimal therapy	35 (9.5%)	6 (2.4%)	< 0.001*	The use of naproxen for headache, which is contraindicated in LT recipients.
Unnecessary drug	216 (58.7%)	43 (17.3%)	0.001^Ω^*	The use of 3 low dosed antihypertensive drugs by a patient with a well-regulated blood pressure.
Untreated indication	25 (6.8%)	7 (2.8%)	0.040*	No anticoagulant prophylaxis, statin and/or ACE inhibitor in a patient with a history of acute coronary syndrome.
Suboptimal dose				
Dose too high	10 (2.7%)	1 (0.4%)	0.057	Long-term use (> 6 months) of apixaban at a dose of 5 mg twice daily instead of 2.5 mg twice daily because of a worse renal function.
Dose too low	23 (6.3%)	1 (0.4%)	< 0.001*	Increase the dose of metformin because of a poorly controlled diabetes mellitus and sufficient renal function.
Dosage regime				
Too frequent	6 (1.6%)	5 (2.0%)	0.763	Magnesium hydroxide usage of 4 times daily while not needed.
Not frequent enough	2 (0.5%)	0 (0.0%)	0.518	Mycophenolate mofetil prescribed once daily instead of twice daily.
Use	6 (1.6%)	60 (24.2%)	< 0.001*	Simplification of complex medication schedules
Question	0 (0.0%)	10 (4.0%)	< 0.001*	Answering a question regarding the use of tacrolimus during pregnancy.
Other	11 (3.0%)	70 (28.2%)	< 0.001^Ω^*	Discrepancies between medication recorded in the patients’ medical records and actual medication used by patient.

Data presented are counts (%) and differences between groups were analyzed using the Fischer’s exact test unless otherwise noted

^Ω^Pearson’s chi-squared test; *indicates a statistical significant difference of p < 0.05

In the non-MC cohort significantly more patients used unnecessary drugs (72.1% *versus* 39.4%, p < 0.001) compared to the MC cohort and 16.5% of the patients in the non-MC cohort used suboptimal doses. The most prevalent unnecessary drugs used were proton pump inhibitors, opioids, benzodiazepines, vitamin D and calcium supplementation. Suboptimal doses were mostly found in patients with poorly controlled diabetes.

A total of 35 LT recipients in the MC cohort had their first MC with the clinical pharmacist, 55 had their second MC and 4 had their third MC. Patients having their first MC with the clinical pharmacist had the most MRPs. The number of MRPs for ADRs in patients having their second MC with the clinical pharmacist was reduced compared to patients having their first MC (16.2% versus 9.7%). The number of MRPs for unnecessary drugs, usage issues and discrepancies in the medication list in patients having their second MC did not differ compared to patients having their first MC.

### Interventions proposed for MRPs

**Fig. 1:**
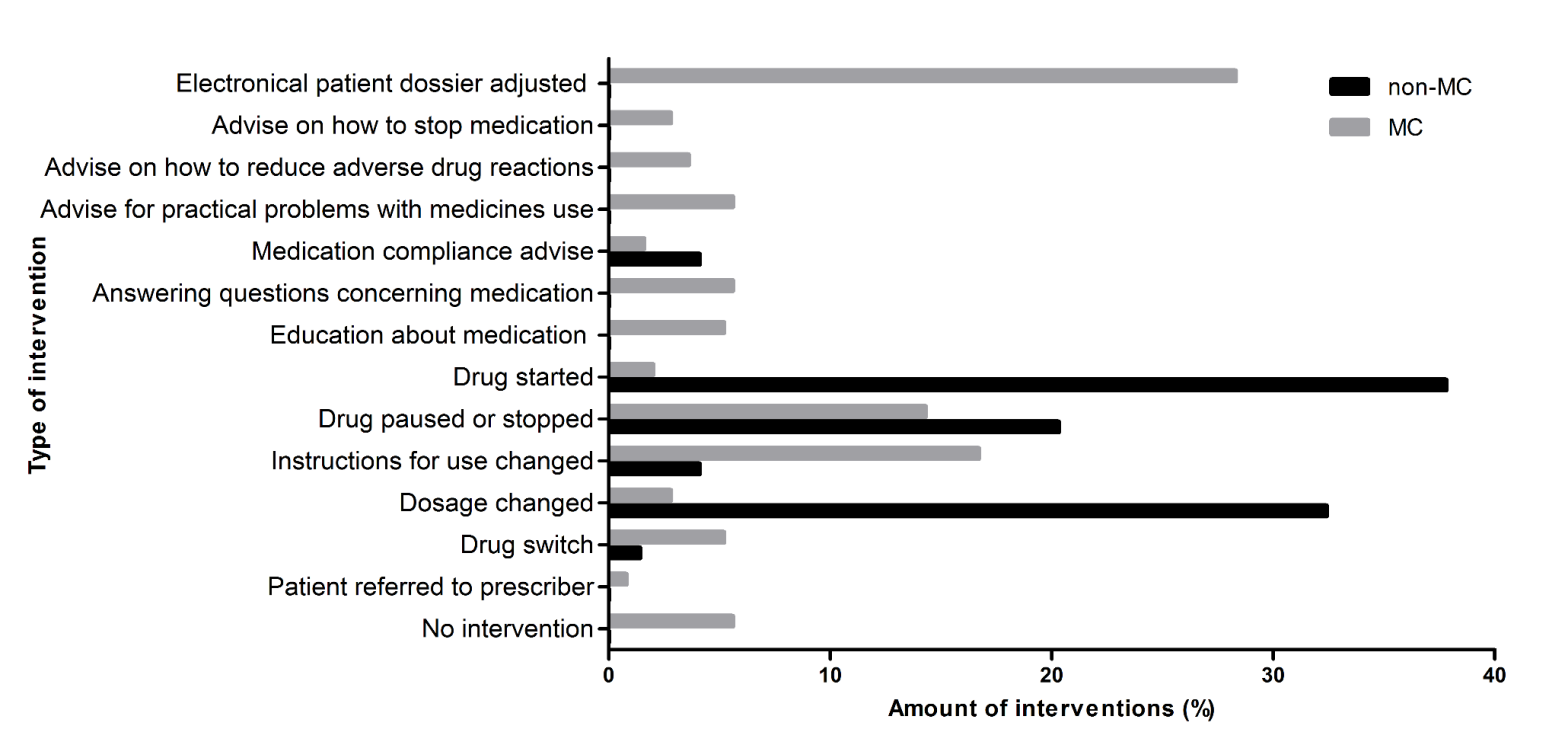
**Interventions proposed for the MRPs (%) in the non-MC and MC cohort.** A total of 74 interventions were proposed by the hepatologist in the non-MC cohort and 251 interventions were proposed by the clinical pharmacist in the MC cohort

Figure 1 shows the interventions proposed for the MRPs. A total of 74 interventions were proposed by the hepatologist in the non-MC cohort (median, 0.0; IQR, 0.0–1.0; maximum 2) and 251 interventions were proposed by the clinical pharmacist in the MC cohort (median, 2.0; IQR, 1.0–4.0; maximum 10). Interventions in the non-MC cohort and MC cohort were carried out by a hepatologist. The most prevalent interventions by a hepatologist were starting a drug (28/74, 37.8%), changing a dose (24/74, 32.4%), and pausing or stopping a drug (15/74, 20.3%). The most prevalent interventions by the clinical pharmacist were adjusting the patient file (71/251, 28.3%), changing the instructions for use (42/251, 16.7%) and pausing or stopping a drug (36/251, 14.3%).

The clinical pharmacist resolved 251 MRPs of which 155 (61.8%) were accepted by the hepatologist and 46 (18.3%) were not accepted by the hepatologist. Examples of accepted interventions were: lowering the dose of magnesium hydroxide and stopping proton pump inhibitors due to the absence of an indication for the high dose and optimizing the dosing regimen. Examples of interventions not accepted were: stopping drugs prescribed by another physician due to the absence of an indication, optimizing the antihypertensive therapy according to the guidelines and changing to another class of laxatives because of taste complaints by the patient. Due to the need for follow-up, it is unknown if 11 (4.4%) interventions were accepted by the LT recipient and due to the nature of the MRP 39 (15.5%) interventions could not be followed up.

### Secondary endpoints: Satisfaction with Information about Medicines and the Medication Adherence

A total of 132 LT recipients participated in the surveys: 84 in the non-MC cohort and 48 in the MC cohort. The completion rate was 80.5%.

Baseline characteristics of participants for the SIMS and MARS-5 surveys are shown in Table 3. The median age differed significantly between the two groups (54.0 years (IQR: 43.0–65.0) in the non-MC cohort versus 63.5 years (IQR: 54.0–68.0) in the MC cohort (p = 0.027)). The majority in both groups were men: 60.7% in the non-MC cohort and 64.4% in the MC cohort.

**Table 3:** Baseline characteristics of the LT recipients for the SIMS and MARS-5 questionnaire

		non-MC cohort (n = 84)	MC cohort (n = 48)	p-value
Age (year (median [IQR])		54.0 (43.0–65.0)	63.5 (54.0–68.0)	0.027^Ω^*
Gender	Male (n,%)	51 (60.7%)	31 (64.6%)	0.552
Education^¥^	None	2 (2.4%)	2 (4.2%)	0.620^∫^
	ISCED 1	3 (3.6%)	4 (8.3%)	0.254^∫^
	ISCED 2	14 (16.7%)	7 (14.6%)	0.810^∫^
	ISCED 3	33 (39.3%)	16 (33.3%)	0.507
	ISCED 6	23 (27.4%)	13 (27.1%)	0.987
	ISCED 7	6 (7.1%)	1 (2.1%)	0.421^∫^
	ISCED 8	0 (0.0%)	3 (6.3%)	0.046^∫^*
	Missing information	3 (3.6%)	1 (2.1%)	-

Data presented are counts (%) and differences between groups were analyzed using the Pearson’s chi-squared test unless otherwise noted

^Ω^Mann-Whitney U-Test; ^∫^Fisher’s Exact Test; *indicates a statistically significant difference of p < 0.05; ^¥^The International Standard Classification of Education 2011 (ISCED 2011) was used to convert the Dutch educational system into an international one. ISCED 4 and 5 does not exist in the Dutch educational system

### Satisfaction with Information about Medicines

Table 4 shows the overall satisfaction and factors associated with the satisfaction of LT recipients with the information about medicines in both cohorts. LT recipients in both cohorts are satisfied with the information about medicines with lower educated LT recipients less satisfied and higher educated LT recipients more satisfied. LT recipients aged < 55 and > 65 years appeared to be more satisfied in the MC cohort compared to the non-MC cohort. LT recipients in the MC cohort were more satisfied compared to the non-MC cohort (72,9% *versus* 64,3%, p = 0.309).

**Table 4:** Overall satisfaction and factors associated with the satisfaction of LT recipients with the information about medicines in the non-MC and MC cohort

		non-MC (n = 84)	MC cohort (n = 48)	
		Satisfied	Satisfied	p-value
Overall satisfaction		54/84 (64.3%)	35/48 (72.9%)	0.309
Gender	Male	33/51 (64.7%)	22/31 (71.0%)	0.558
	Female	21/33 (63.6%)	12/16 (75.0%)	0.426
	Missing information	-	1/1 (100.0%)	-
Age	< 55 years	26/41 (63.4%)	11/12 (91.7%)	0.055
	55–65 years	15/20 (75.0%)	9/17 (52.9%)	0.044
	> 65 years	9/18 (50.0%)	14/17 (82.4%)	0.161
	Missing information	4/5 (80.0%)	1/2 (50.0%)	-
Education*	Low	32/52 (61.5%)	20/29 (69.0%)	0.585
	High	20/29 (69.0%)	13/17 (76.5%)	0.504
	Missing information	2/3 (66.7%)	2/2 (100.0%)	-

*Patients are considered highly educated for ISCED classifications of ≥ 6 and low educated for ISCED classifications of < 6

LT recipients in both cohorts were less satisfied with the information about the mechanism of action, ADRs and whether the medicine interferes with other drugs (supplementary Table 2).

### Medication Adherence

LT recipients in both cohorts had a median MARS-5 score of 24.0 (IQR: 24.0–25.0), which indicates a high medication adherence in both cohorts.

## Discussion

To our knowledge, this is the first study describing the impact of a clinical pharmacist by investigating the differences in MRPs in LT patients with and without an MC during an outpatient annual check-up. LT recipients in the MC cohort had significantly fewer MRPs as using of unnecessary drugs, wrong drugs/suboptimal therapy, and having dosing issues. In the MC cohort significantly more MRPs were identified as unintentional nonadherence, problems in drug use, questions regarding the drugs, and other problems. We demonstrated a high prevalence of MPRs in LT recipients in the outpatient setting with a median of 1 MRP in the non-MC cohort and 2 MRPs in the MC cohort. The most frequently reported MRPs in both cohorts were unnecessary drug use, problems in drug use and ADRs. Most LT recipients in both cohorts were satisfied with the information about medicines.

Our findings are in line with the results of Flamme-Obry et al. They showed that an interview with the clinical pharmacist at discharge could help to reduce MRPs in kidney transplant recipients [17]. Flamme-Orby found that their intervention resulted in fewer MRPs as interactions between drugs, ADRs and wrong usage of medicines. By introducing a medication consultation by a clinical pharmacist we resolved a substantial number of MRPs as the wrong usage of medicines. Furthermore, several studies showed comparable results to ours with regards to the most prevalent MRPs [10, 18]. The most prevalent MRPs in these studies were ADRs, nonadherence issues, and the use of unnecessary drugs. By detecting and preventing the use of unnecessary drugs the clinical pharmacist can contribute to relevant social issues as preventing for unnecessary healthcare costs and the sustainable use of medicines.

Most frequently proposed interventions by the clinical pharmacist in the MC cohort were instructions for use changed, drug paused or stopped and patient file adjusted. Cohen et al. showed that a pharmacist-driven medication reconciliation significantly reduced medication discrepancies [19]. Furthermore, another study by Ho et al., showed that pharmacists working as part of the multidisciplinary transplant team identified and resolved medication discrepancies and thereby improved the medication safety at a transplant clinic [20]. Interestingly, in both studies the average number of discrepancies per patient was higher compared to our study. This might been explained by the fact that in the Netherlands medication reconciliation is mandatory for every patient admitted to a hospital. The most frequently proposed interventions by the hepatologist in the non-MC cohort were the start/stop of a drug or the change of the dosage of a drug. This is a consequence of the organization of the Dutch healthcare system in which only doctors are allowed to initiate those interventions. Interventions not accepted by the hepatologist were mostly due to the fact that these drugs were prescribed by other prescribers or patients were not willing to change/stop their medication.

Most LT recipients in both cohorts were satisfied with the information about medicines. In the non-MC cohort, patients indicated that they were less satisfied with the information regarding the mechanism of action, ADRs and possible interactions. This might be explained by the fact that hepatologists focus less on educating patients about their medicines and ADRs. These findings are in line with Klewitz et al. who found a high prevalence of dissatisfaction with information about medication, specifically ADRs, in kidney transplant recipients [16]. A study evaluating the SIMS in patients using anticancer agents also showed comparable results to our non-MC cohort with patients dissatisfied with the information concerning the mechanism of action of drugs, the risk of ADRs, and the interference with other drugs [21]. Based on the results in this study, educating patients about their medication, ADRs and dosing regimens is not commonly done but is important to prevent for MRPs.

Strengths of our study are the real-life clinical setting and the fact that we included a representative part, almost 50%, of the LT recipients being seen at the outpatient clinic. A limitation of our study is the fact that in the non-MC cohort, MRPs and interventions were detected using the patients’ medical records, including the patients’ medication list, laboratory results and medical history. The clinical pharmacist did not attend the consultations by the hepatologist. Whereas patients in the MC cohort had an actual MC with the clinical pharmacist. As a consequence, other types of and less MRPs were detected in the non-MC cohort since less information was available. Another limitation is the fact that we did not investigate the impact of the MRPs in relation to potential harm and clinical outcomes. Further research is needed to study the cost-effectiveness of the MCs carried out by a clinical pharmacist. In addition, evaluating the responsibilities and mandate of a clinical pharmacist to resolve MRPs caused by drugs prescribed by different physicians is needed to optimize the medication safety in patients with multiple comorbidities.

## Conclusion

LT recipients in the MC cohort had significantly fewer MRPs as the usage of unnecessary drugs, wrong drugs/suboptimal therapy, and having dosing issues. Over 70% of the patients in the non-MC cohort were using unnecessary drugs. LT recipients in both cohorts are satisfied with the information about medicines. Since clinical pharmacists bring different perspectives to post-transplant care, including a clinical pharmacist in the multidisciplinary transplant team has an added value for improving the pharmaceutical care and optimizing medication safety in these patients.

## Electronic supplementary material

Below is the link to the electronic supplementary material.

Supplementary Material 1
