# Supplementary material for: Evaluation of medication-related problems in liver transplant recipients with and without an outpatient medication consultation by a clinical pharmacist: a cohort study
Source: Int J Clin Pharm. 2022 Sep 13;44(5):1114–22. doi: 10.1007/s11096-022-01423-6 (PMC9618499; doi:10.1007/s11096-022-01423-6)
Supplement: Supplementary file 1 — Supplementary Material 1 [file 11096_2022_1423_MOESM1_ESM.docx]

**Supplementary table 1**. **MRP classification according to the classification of the Pharmaceutical Care Network Europe version 9.0. (5)**

| Category | Subclassification | Definition |
| --- | --- | --- |
| Nonadherence | Intentional | The patient uses/takes intentionally less drug than prescribed or does not take the drug at all. |
|  | Unintentional | The patient uses/takes unintentionally less drug than prescribed or does not take the drug at all. |
| Adverse drug reactions |  | The patient has a medical problem that is the result of an adverse drug reaction. |
| Drug interaction |  | The patient has a medical problem that is the result of a drug-drug, drug-food, or drug-laboratory interaction. |
| Indication | Wrong drug | The patient has a drug indication but is taking the wrong drug. |
|  | Unnecessary drug | The patient is taking a drug for no medically valid indication. |
|  | Untreated indication | The patient has a medical problem that requires drug therapy but is not receiving a drug for that indication. |
| Suboptimal dose | Dose too high | The patient has a medical problem that is being treated with too much of the correct drug. |
|  | Dose too low | The patient has a medical problem that is being treated with too little of the correct drug. |
| Dosage regime | Too frequent | The patient has a medical problem that is being treated with the correct drug of which the dosage regime is too frequent. |
|  | Not frequent enough | The patient has a medical problem that is being treated with the correct drug of which the dosage regime is not frequent enough. |
| Use |  | The patient administers/uses the drug in a wrong way. |
| Question |  | The patient has questions concerning the medication. |
| Other |  | The patients’ medication list is incomplete or has discrepancies. |

**Supplementary table 2. Detailed information regarding the degree of satisfaction of the LT recipients with the various aspects of information about medicines.**

|  | non-MC cohort (n=84) | | | | | MC cohort (n=48) | | | | | p-value |
| --- | --- | --- | --- | --- | --- | --- | --- | --- | --- | --- | --- |
|  | *Satisfied (%)* | | *Not satisfied (%)* | | | *Satisfied (%)* | | *Not satisfied (%)* | | |  |
|  | About right | None needed | Too much | Too little | None received | About right | None needed | Too much | Too little | None received |  |
| What your medicine is called. | 90.5 | 2.4 | 2.4 | 3.6 | 1.2 | 79.2 | 12.5 | 4.2 | 2.1 | 2.1 | 0.177 |
| What your medicine is for. | 89.3 | 2.4 | 2.4 | 3.6 | 2.4 | 87.5 | 8.3 | 2.1 | 2.1 | 0 | 0.438 |
| What it does. | 86.9 | 1.2 | 2.4 | 7.1 | 1.2 | 89.6 | 4.2 | 2.1 | 4.2 | 0 | 0.589 |
| How it works. | 78.6 | 1.2 | 2.4 | 10.7 | 7.1 | 87.5 | 2.1 | 2.1 | 6.3 | 2.1 | 0.624 |
| How long it will take to act. | 69.0 | 4.8 | 2.4 | 14.3 | 9.5 | 77.1 | 12.5 | 0 | 8.3 | 2.1 | 0.125 |
| How you can tell if it is working. | 61.9 | 6.0 | 1.2 | 14.3 | 16.7 | 68.8 | 12.5 | 2.1 | 10.4 | 6.3 | 0.300 |
| How long you will need to be on your medicine. | 83.3 | 9.5 | 4.8 | 1.2 | 1.2 | 83.3 | 10.4 | 2.1 | 2.1 | 2.1 | 0.921 |
| How to use your medicine. | 94.0 | 3.6 | 2.4 | 0 | 0 | 85.4 | 8.3 | 2.1 | 2.1 | 2.1 | 0.280 |
| How to get a further supply. | 88.1 | 4.8 | 0 | 3.6 | 3.6 | 70.8 | 16.7 | 4.2 | 6.3 | 2.1 | 0.039 |
| Whether the medicine has any unwanted effects (side effects). | 77.4 | 2.4 | 0 | 16.7 | 3.6 | 77.1 | 8.3 | 0 | 14.6 | 0 | 0.243 |
| What are the risks of you getting side effects. | 67.9 | 2.4 | 0 | 15.5 | 14.3 | 75.0 | 8.3 | 0 | 12.5 | 4.2 | 0.125 |
| What you should do if you experience unwanted side effects. | 67.9 | 4.8 | 2.4 | 14.3 | 10.7 | 60.4 | 16.7 | 2.1 | 14.6 | 6.3 | 0.222 |
| Whether you can drink alcohol whilst taking this medicine. | 71.4 | 15.5 | 2.4 | 3.6 | 7.1 | 56.3 | 31.3 | 0 | 6.3 | 6.3 | 0.177 |
| Whether the medicine interferes with other medicines. | 64.3 | 6.0 | 1.2 | 19.0 | 9.5 | 56.3 | 18.8 | 2.1 | 10.4 | 12.5 | 0.142 |
| Whether the medication will make you feel drowsy. | 60.7 | 10.7 | 0 | 13.1 | 15.5 | 64.6 | 14.6 | 0 | 12.5 | 8.3 | 0.644 |
| Whether the medication will affect your sex life. | 36.9 | 15.5 | 0 | 15.5 | 32.1 | 43.8 | 22.9 | 4.2 | 14.6 | 14.6 | 0.076 |
| What you should do if you forget to take a dose. | 71.4 | 7.1 | 1.2 | 8.3 | 11.9 | 72.9 | 10.4 | 0 | 10.4 | 6.3 | 0.715 |
